# Assessment of vascular function in individuals with hyperglycemia: a cross-sectional study of glucose – induced changes in digital volume pulse

**DOI:** 10.1186/s40200-015-0153-2

**Published:** 2015-04-10

**Authors:** Fariba Alaei-Shahmiri, Yun Zhao, Jill Sherriff

**Affiliations:** School of Public Health, Curtin University, Kent Street, Bentley, Perth, Western Australia; Iran University of Medical Sciences (IUMS), Tehran, Iran

**Keywords:** Hyperglycemia, Arterial stiffness, Digital volume pulse analysis, Stiffness index, Reflection index, Photoplethysmography

## Abstract

**Background:**

Arterial stiffness is an independent risk factor for cardiovascular disease and its progression may be accelerated in the presence of hyperglycemia, either fasting or postprandial. The current study assessed vascular function in subjects with pre-diabetes hyperglycemia, using digital volume pulse analysis technique.

**Methods:**

We conducted a cross-sectional study examining vascular function in the fasting and postprandial (glucose-induced) state in 44 adults, consisting of 17 subjects with pre-diabetic hyperglycemia and 27 normoglycemic volunteers. Photoplethysmography of the digital volume pulse (DVP) was used to determine stiffness index (SI) and reflective index (RI), as main measures of larger artery stiffness and vascular tone, respectively.

**Results:**

Our results showed a significantly higher (Ln) fasting SI in the hyperglycemic group compared with the control group (2.19 ± 0.32 vs. 1.96 ± 0.22, P = 0.005). However, this pattern reversed after adjustment for potential confounders. In multiple linear regression analysis, (Ln) SI was related to age (β = 0.01, 95% CI: 0.01-0.02, P < 0.001) and systolic blood pressure (SBP) (β = 0.01, 95% CI: 0.00-0.01, P < 0.05), but not with W/H, diastolic blood pressure (DBP), fasting plasma glucose (FPG) or serum lipids. Furthermore, age (β = 0.02, 95% CI: 0.01-0.03, P < 0.001) and mean arterial pressure (MAP) (β = 0.01, 95% CI: 0.00-0.02, P < 0.05) were found as the strong predictors of fasting SI in hyperglycemic group. Neither FPG nor 2-h plasma glucose was a significant predictor for SI in hyperglycemic group, after accounting for age and MAP. Subjects with hyperglycemia had a 15% blunted change in postprandial AUCs for RI, adjusted for the respective baseline measurements (−9.40 ± 3.59 vs. -11.00 ± 2.84%) but these did not attain statistical significance.

**Conclusion:**

Increased arterial stiffness in pre-diabetic subjects is strongly associated with age and MAP. The increased DVP-derived SI reported in patients with pre-diabetic hyperglycemia may result from different frequently accompanied risk factors not just glycemic changes in this range.

## Background

Arterial stiffness is a general term used to define the stiffening of the arterial wall, resulting in a decreased elasticity of the blood vessel. Although the pathogenesis of arterial stiffness is not fully understood, changes in the structure of the extracellular matrix, endothelial function and vascular smooth muscle tone have been implicated in its aetiology [1]. Arterial stiffness is an independent risk factor for cardiovascular disease and occurs mainly as a pathological consequence of ageing [2]. However, its progression may be accelerated in the presence of other risk factors, including hyperglycemia in either diabetic or pre-diabetic ranges [3,4]. Hyperglycemia, either fasting or postprandial, has been shown to enhance arterial stiffness through several mechanisms, causing endothelial dysfunction as well as alternations in the structure of vascular extracellular matrix [5-7].

Arterial stiffness can be evaluated by a variety of different techniques. Pulse Trace PCA2 is a non- invasive device used to assess vascular stiffness and endothelial function by recording digital volume pulse (DVP). In comparison with other available methods for arterial stiffness assessment, pulse trace system is technically the simplest, independent of operator skill, and validated in different setting and diseases [8-10]. Also, its reproducibility is consistent with other accepted methods [11]. Pulse Trace system analyses DVP waveform to determine two main measures of vascular function: stiffness index (SI) and reflection index (RI).

The SI derived from DVP is an accepted measure of large artery stiffness and correlates strongly with the central pulse wave velocity (PWV, the ‘gold standard’ arterial stiffness parameter) as well as the augmentation index determined by pulse wave analysis (PWA) method [12]. SI derived from DVP was designed as a potent discriminator of increasing cardiovascular complications in patients with cardiovascular risk factors including smoking, hypertension, diabetes, hypercholesterolemia and central obesity [13]. However, the clinical utility of this method in screening arterial stiffness in individuals with hyperglycemia at pre-diabetic ranges is not well known. The RI derived from DVP was suggested as a reliable parameter reflecting the vascular tone of small arteries [14]. Furthermore, response of RI to endothelium- dependent vasoactive agents has been established as a useful measure of endothelial function in individuals with impaired glucose metabolism, hypertension and coronary heart disease [8,15,16] that depends on endothelial-derived nitric oxide (NO) [8]. It was shown that postprandial hyperglycemia induced by oral glucose loading alters endothelial function even in normal individuals [17,18]. Accordingly, the temporary impairment of endothelium-dependent vasorelaxation observed following an oral glucose challenge has been already considered as a measure to assess endothelial function in individuals with hyperglycemia using other techniques including the flow-mediated vasodilation (FMD) assessment [19]. However, there has been no published literature investigating the changes in RI after a glucose challenge in subjects with hyperglycemia. Given these gaps, the objective in this study was to assess vascular function in the fasting and postprandial (glucose-induced) states in subjects with pre-diabetes hyperglycemia, using this technique.

## Material and methods

### Subjects

Forty four adults consisting of seventeen subjects with pre-diabetic hyperglycemia and twenty seven normal healthy volunteers, with BMI 27.8 ± 4.4 kg/m^2^ and aged 44.4 ± 15.2 years were recruited from the community. Pre-diabetics were diagnosed by a fasting plasma glucose level ≥ 6.1 mmol/l (110 mg/dl) and < 7.0 mmol/l (126 mg/dl), and/or 2-h oral glucose tolerance test value ≥ 7.8 mmol/l (140 mg/dl) and < 11.1 mmol/l (200 mg/dl) [20]. Exclusion criteria were: smoking, known impaired renal or liver function, females who were pregnant or lactating, hypo- and hyperthyroidism and known diabetes mellitus. No subject was on any medication which could affect blood pressure, vascular function or metabolic outcomes, and no subject had a albumin/creatinine ratio ≥ 300 mg/g representing clinical proteinuria [21]. This study was conducted according to the principal of the Declaration of Helsinki and approved by the Curtin University Human Research Ethics Committee (Approval number HR 118/2008).

### Study design and procedure

This cross-sectional study evaluated fasting and arterial stiffness in individuals with impaired glucose regulation relative to normal healthy volunteers. Following the initial screening, eligible volunteers were informed about the study’s protocol in detail during a short introductory visit, and written consent was obtained from the subjects. Participants were required to be on an unrestricted diet that had at least 150–200 g of carbohydrate for the three days prior to the test, and to consume a standard meal provided by investigators on the evening before the clinical day. They were also asked to refrain from alcohol and strenuous exercise for the 24 h before the visit. On the morning of the clinical day, subjects attended the out-patient clinic, School of Public Health, Curtin University, following an overnight fast of 10–12 hours. Anthropometric measurements were taken with subjects dressed in a gown with no shoes and empty bladder. Participants were then asked to rest 30 minutes prior to the measurements of blood pressure and vascular function, and blood sampling. Fasting blood samples were collected via venipuncture into serum and plasma tubes. Aliquots of separated plasma and serum were frozen at −80°C until analysed. Following fasting measurements, a 75-g oral glucose load was administrated to the subjects. The second blood sample was taken 2 hours after the test load.

### Measurements of arterial blood pressure

After a mandatory rest period of 30 minutes, systolic and diastolic blood pressures (SBP & DBP) were measured on subjects’ right arms in supine position, using a vital signs patient monitor (Cardiocap II, Datex, Helsinki, Finland) with a standard cuff for adults. Three blood pressure measurements, with 2-minutes intervals, were recorded in the fasting state and the average value was reported.

### Measurements of vascular function

#### Calculation of the indices derived from digital volume pulse

**Figure 1:**
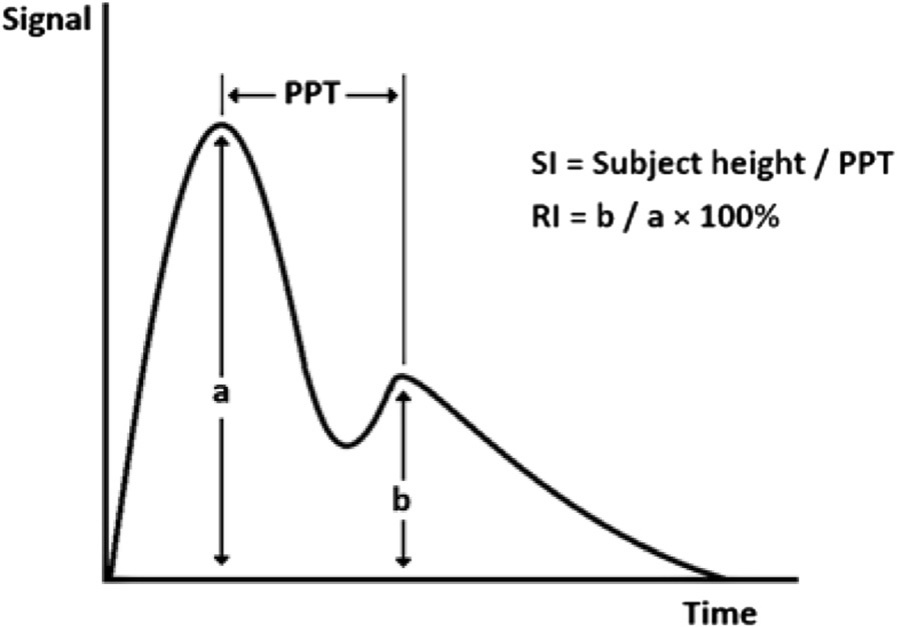
**Indices derived from digital volume pulse analysis.** SI, stiffness index; RI, reflection index; PPT, peak-to-peak time.

The technique of DVP is based on measuring infra-red light transmission through the finger (photoplethysmography) [8]. Photoplethysmography of DVP is used to determine stiffness index (SI) and reflective index (RI), as main measures of larger artery stiffness and vascular tone of small arteries, respectively. RI is computed by the% ratio of the heights (amplitude) of reflected wave (diastolic component) to the systolic peak, and is a measure of pulse wave reflection and the small artery tone. SI can be calculated from the subject’s height divided by the time between systolic and diastolic peaks, and is a marker of large arterial stiffness [Figure 1] [11].

#### Arterial stiffness measurement protocol

Vascular function was assessed by a Pulse Trace machine (PCA2, Micro Medical Ltd., UK), recording RI and SI as the main indices determined from DVP analysis. photoplethysmography sensor transmitting infra-red light was placed on the index finger of subjects. The digital volume pulse measurements were recorded with subjects supine in a temperature-controlled room (22 ± 2°C). Two readings, at least 2 minutes apart, were taken in the fasting state by the same researcher. Each of these measurements was the average of three recordings with intervals of 30 seconds. Within-subject coefficients of variation were 8.01% and 6.03% for SI and RI measurements, respectively. Subsequent measurements were made serially at 30, 60, 90 and 120 minutes after ingestion of the test load. The changes in postprandial RI were determined by subtracting the total area under the RI curve between 0 and 2 h from the area below the baseline measurements, representing the change in the area for RI following consumption of glucose load.

### Biochemical measurements

The stored blood samples were analysed for markers of metabolic control including: fasting and 2-h plasma glucose, fasting and 2-h insulin, serum lipid profile (triglyceride, total cholesterol, HDL-cholesterol and LDL-cholesterol) and serum high-sensitivity C-reactive protein (hs-CRP). Plasma glucose levels were determined with the hexokinase method. Insulin levels were measured by the Architect insulin assay. The results of fasting plasma glucose and insulin were also used to estimate the hemostatic model assessment (HOMA-IR) score, as an index of insulin resistance [22]. Enzymatic colorimetric assays were used to determine the levels of serum triglyceride and total cholesterol. Concentrations of HDL-cholesterol were directly measured in the serum samples by the Ultra HDL assay. Glucose, insulin and lipid profile analyses were performed using the Abbott diagnostic kits (Abbott Laboratories, IL, USA) with a within- and between-run coefficient of variation of < 4.3%. LDL-cholesterol was calculated using a modified version of Friedewald equation [23] with quantities in mmol/l. Serum hs-CRP was measured by nephelometry using a BNII system (Siemens Healthcare Diagnostic inc. Newwark, DE, USA) with between- and within-run coefficient of variation of 8.35% and 5.7%, respectively.

### Statistical analysis

All statistical analyses were performed using IBM SPSS Statistics for windows (Version 19.0. IBM Corp. Released 2010. Armonk, NY: IBM Corp USA). An independent samples t-test and a Mann–Whitney U test were used to determine the significances of differences in mean and median of the clinical measurements between study groups, respectively. Correlation analysis was done by calculating Pearson correlation coefficient (r) and Spearman’s rank correlation coefficient (ρ). Multiple linear regression analyses were carried out to explore the significant predictors of being arterial stiffness as measured by stiffness index (SI) and reflective index (RI), for all subjects and for hyperglycemic group, respectively. All tests were two-tailed and a P < 0.05 was considered as statistically significant.

## Results

The characteristics of hyperglycemic subjects [10 males, 7 females] and normal control volunteers [11 males, 16 females] participating in this study are presented in Table 1. Participants were classified as normal or hyperglycemic based on World Health Organisation criteria [20], using venous plasma glucose measurements (FPG & 2-h GTT). There were seventeen hyperglycemic subjects consisting of twelve cases of impaired glucose tolerance (IGT) [six cases with pure IGT and six cases with both elevated fasting and 2-h plasma glucose] and five cases of impaired fasting glucose (IFG). Subjects in the hyperglycemic group were significantly older than normal healthy volunteers (median: 60 y, IQR: 18 vs. median: 34 y, IQR: 15; P < 0.001) and had greater waist circumference (100.38 ± 11.47 cm vs. 88.70 ± 12.28 cm; P = 0.003), waist/hip ratio (median: 0.93, IQR: 0.12 vs. median: 0.81, IQR: 0.13; P = 0.001), but similar BMI (normal group: 27.32 ± 4.60 kg/m^2^, hyperglycemic group: 28.50 ± 4.07 kg/m^2^). Both systolic and diastolic blood pressure in the hyperglycemic group were significantly higher than those of normal participants (P < 0.001). There also were significant differences in fasting and 2-h plasma glucose levels between study groups (P <0.001) [Table 1].

**Table 1:** **Characteristics of participants in the study groups**

	**Hyperglycemic group (n = 17)**	**Normal control group (n = 27)**	**p- value**
Age (y)	60 (18.00)	34 (15.00)	<0.001
BMI (kg/m^2^)	28.50 ± 4.07	27.32 ± 4.60	0.40
WC (cm)	100.38 ± 11.47	88.70 ± 12.28	0.003
W/H	0.93 (0.12)	0.81 (0.13)	0.001
SBP (mmHg)	121.24 ± 10.77	106.74 ± 9.50	<0.001
DBP (mmHg)	72.82 ± 8.70	62.96 ± 6.11	<0.001
Fasting glucose (mmol/l)	6.10 (0.55)	5.00 (0.50)	<0.001
2-h glucose (mmol/l)	8.35 ± 1.16	6.13 ± 0.93	<0.001
Fasting insulin (μIU/ml)	6.5 (6.85)	5.7 (3.60)	0.20
2-h insulin (μIU/ml)	74.80 (66.05)	52.10 (26.40)	0.09
HOMA-IR	1.74 (2.00)	1.32 (1.00)	0.04
TG (mmol/l)	1.23 (0.79)	1.27 (0.93)	0.35
Total chol. (mmol/l)	5.17 ± 0.66	4.97 ± 1.12	0.50
LDL-chol. (mmol/l)	3.22 ± 0.57	2.99 ± 0.89	0.36
HDL-chol. (mmol/l)	1.22 ± 0.22	1.24 ± 0.28	0.83
hs-CRP (mg/l)	1.41 (2.72)	0.88 (1.69)	0.56

Data are expressed as mean ± SD or as median (IQR) for skewed data.

**Figure 2:**
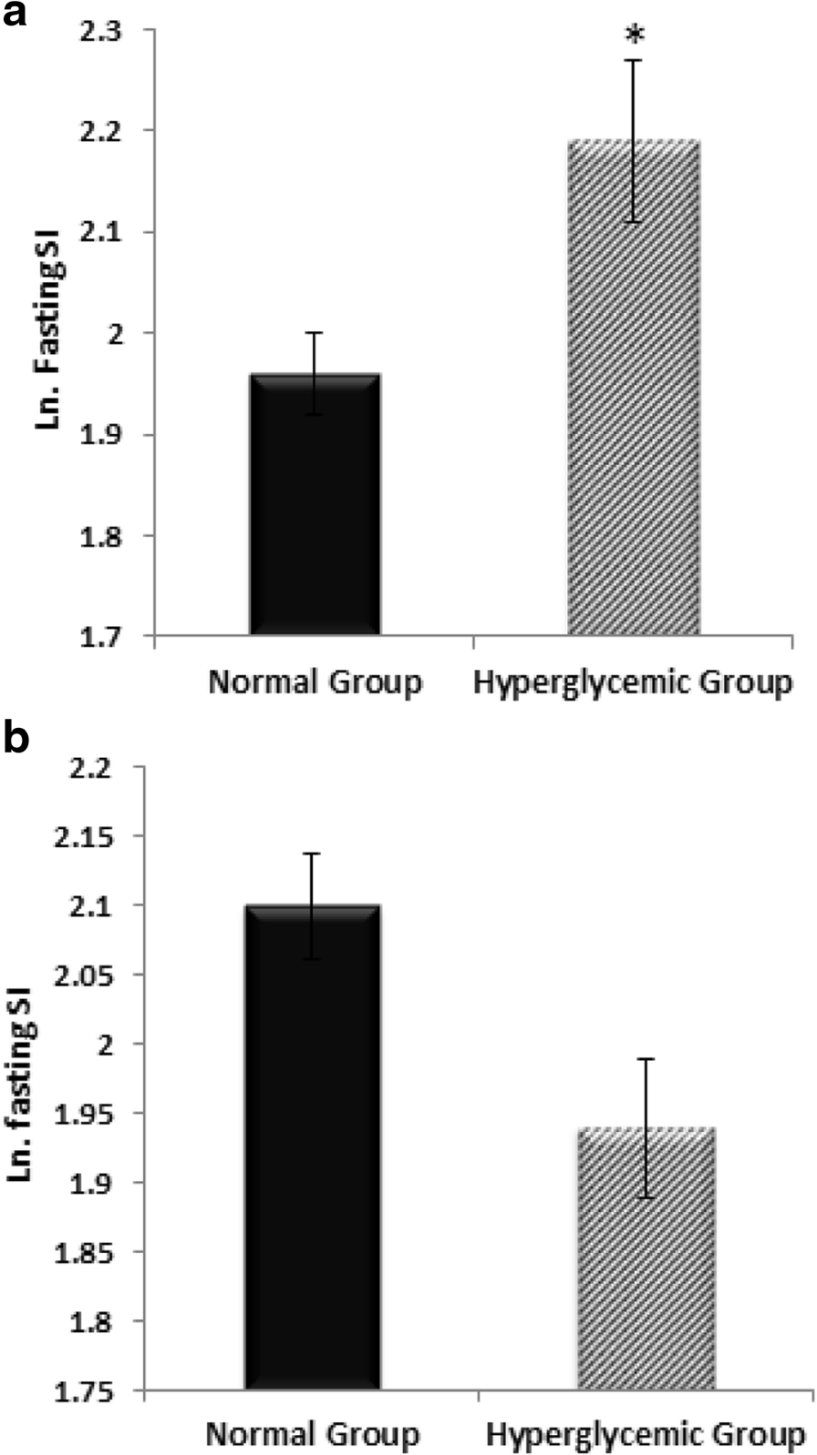
**Log-transformed fasting SI without adjustment (a); and Log-transformed fasting SI, after adjusting for age, sex, SBP, DBP and W/H, (b) determined in normal and hyperglycemic groups.** Data are presented as mean ± SEM. *, p < 0.01 compared to the normal control group.

The mean value (±SD) of (Ln) fasting SI measured in the hyperglycemic group was significantly higher than that of the normoglycemic group (2.19 ± 0.32 vs. 1.96 ± 0.22, P = 0.005). However, this pattern changed after adjustment for potential confounders (age, sex, SBP, DBP and W/H ratio) [Figure 2].

Fasting SI measured in all subjects was significantly correlated with age (ρ = 0.78, P < 0.001), FPG (ρ = 0.54, P < 0.001), SBP (ρ = 0.62, P < 0.001), DBP (ρ = 0.47, P = 0.001), pulse pressure (PP, ρ = 0.44, P < 0.01), mean arterial pressure (MAP, ρ = 0.58, P < 0.001), WC (ρ = 0.35, P = 0.02) and W/H ratio (ρ = 0.55, P < 0.001). The analysis performed in the study groups showed that in the hyperglycemic group, fasting SI was significantly correlated with age (ρ = 0.87, P < 0.001), FPG (ρ = 0.56, P = 0.02), SBP (ρ = 0.56, P = 0.02), mean arterial pressure (ρ = 0.50, P < 0.05) and W/H ratio (ρ = 0.57, P = 0.02). However, fasting SI in the normoglycemic group remained correlated only with age (ρ = 0.63, P < 0.001), mean arterial pressure (ρ = 0.40, P < 0.05) and SBP (ρ = 0.48, P = 0.01). There was no significant correlation between fasting SI and lipid profile (P values > 0.05) [Table 2]. In the multiple linear regression analysis for all subjects, (Ln) fasting SI was positively related to age (β = 0.01, 95% CI: 0.01-0.02, P < 0.001) and SBP (β = 0.01, 95% CI: 0.00-0.01, P < 0.05), but not with W/H, DBP, FPG or serum lipids. Furthermore, age (β = 0.02, 95% CI: 0.01-0.03, P < 0.001) and mean arterial pressure (β = 0.01, 95% CI: 0.00-0.02, P < 0.05) were the strong predictors of fasting SI determined in hyperglycemic group. Neither FPG nor 2-h PG were significant predictors for fasting SI, after accounting for age and MAP in this group.

**Table 2:** **Correlations between DVP-derived indices and clinical characteristics of participants in overall and in the study groups**

**Characteristics**	**Hyperglycemic group**			**Normoglycemic group**			**All subjects**		
	**F.SI**	**F.RI**	Δ **PP. RI**	**F.SI**	**F.RI**	Δ **PP. RI**	**F.SI**	**F.RI**	Δ **PP. RI**
Age (y)	0.87^c^	0.81^c^	0.52^a^	0.63^c^	−0.008	−0.06	0.78^c^	0.34^a^	0.16
BMI (kg/m^2^)	−0.12	0.02	0.02	0.25	0.03	0.26	0.17	0.04	0.19
WC (cm)	0.14	0.26	0.31	0.22	0.04	0.25	0.35^a^	0.16	0.26
W/H	0.57^a^	0.48	0.55^a^	0.33	0.09	−0.11	0.55^c^	0.23	0.09
SBP (mmHg)	0.56^a^	0.56^a^	0.18	0.48^b^	0.08	0.12	0.62^c^	0.3	0.14
DBP (mmHg)	0.34	0.4	0.15	0.3	−0.02	0.03	0.47^b^	0.21	0.09
PP (mmHg)	0.35	0.30	0.08	0.31	0.15	0.15	0.44^b^	0.24	0.14
MAP (mmHg)	0.50^a^	0.50^a^	0.18	0.40^a^	0.02	0.07	0.58^c^	0.26	0.11
FPG (mmol/l)	0.56^a^	0.42	0.32	0.35	0.03	−0.12	0.54^c^	0.2	0.05
2-h PG (mmoI/l)	−0.44	−0.46	−0.13	0.51^b^	0.08	0.19	0.34^a^	−0.01	0.07
HOMA-IR	0.03	0.03	0.16	0.15	−0.23	0.1	0.22	−0.002	0.13
TG (mmol/l)	0.01	−0.12	0.21	0.32	0.01	0.12	0.25	−0.02	0.15
Total Chol. (mmol/l)	−0.03	−0.08	−0.07	0.23	0.14	0.1	0.2	0.09	0.06
HDL-Chol. (mmol/l)	−0.05	−0.01	0.06	0.08	0.25	−0.19	0.05	0.16	−0.12
LDL-Chol. (mmol/l)	−0.19	−0.11	−0.23	0.23	0.08	0.14	0.16	0.04	0.05
hs-CRP(mg/l)	−0.34	−0.23	−0.05	0.35	0.12	0.14	0.12	−0.14	0.12

Data are expressed as correlation coefficient; F.SI, Fasting RI; Δ PP. RI, Change in postprandial AUC for RI from the baseline; PP, pulse pressure; MAP, Mean arterial pressure; ^a^p < 0.05, ^b^p < 0.01, ^c^p < 0.001.

**Figure 3:**
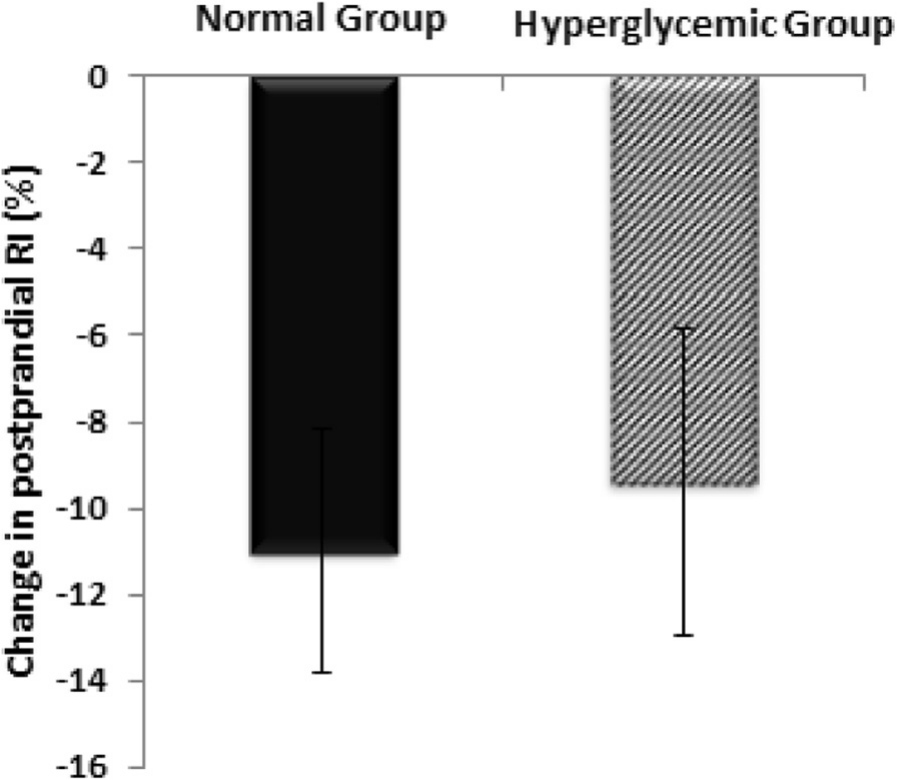
**Changes in the post-glucose challenge RI observed in normal and hyperglycemic groups.** Data are presented as mean ± SEM of the change in the postprandial AUC from the baseline, and are adjusted for the baseline measurements. *, p < 0.05 compared to the normal control group.

Hyperglycemic and normoglycemic groups had mean (±SD) fasting RI 71.62% ± 10.62% and 68.96% ± 10.50%, respectively. In comparison with the normoglycemic group, subjects with hyperglycemia had a 15% blunted change in postprandial AUCs for RI, adjusted for the respective baseline measurements (−9.40 ± 3.59 vs. -11.00 ± 2.84%), though these did not attain statistical significance (P > 0.05) [Figure 3].

There was a significant correlation between age and the fasting RI in overall (r = 0.34, P < 0.05) and in hyperglycemic group (r = 0.81, P < 0.001), but not in normoglycemic group. Fasting RI determined in hyperglycemic group was also significantly correlated with SBP (r = 0.56, P < 0.05) and mean arterial pressure (r = 0.50, P < 0.05). Also, there was no significant correlation between the change in postprandial RI and age or other CVD risk factors assessed in all participants in this study (P > 0.05). However, the change in postprandial RI in hyperglycemic group was significantly correlated with age (r = 0.52, P < 0.05) and W/H ratio (r = 0.55, P < 0.05) [Table 2]. In the multiple linear regression analysis for all participants, there was no significant relationship between different clinical characteristics of the study subjects and fasting RI or the change in postprandial AUC for RI. However, age was found as a significant predictor of fasting RI where the analyses were performed in the hyperglycemic group (β = 0.67, 95% CI: 0.40-0.94, P < 0.001). Furthermore, a regression model including gender (Female: β = −17.05, 95% CI: −32.34--1.76, P = 0.03), WC/HC (β = 169.49, 95% CI: 58.41-280.58, P = 0.007), FPG (β = −30.05, 95% CI: −51.39--8.7, P = 0.01), total cholesterol (β = 27.16, 95% CI: 6.83-47.48, P = 0.01), LDL-cholesterol(β = −42.82, 95% CI: −66.92--18.71, P = 0.003), 2-h PG (β = 8.7, 95% CI: 3.20-14.22, P = 0.005) could predict 72.5% of variability in the change in postprandial AUC for RI in the hyperglycemic group. These variables also remained as significant predictors after adding the respective baseline measurements (P > 0.05) to the model.

## Discussion

It is known that hyperglycemia in either diabetic or pre-diabetic range (IFG, IGT) is associated with a high risk of CVD and mortality [24-26]. While, exact mechanisms through which hyperglycemia increases cardiovascular risk have not been fully elucidated, increased central and peripheral arterial stiffness has been demonstrated as a potential explanation [3]. Aggravated arterial stiffness has been evident in individuals with IFG and IGT, in which arterial stiffness has mainly been assessed using PWV [27-29]. The SI derived from DVP has been reported to be significantly higher in patients with CVD risk factors including diabetics [13]. However, the clinical utility of this technique in screening arterial stiffness in individuals with pre-diabetic hyperglycemia is not well established. The current study is among the first [15,30] investigating arterial stiffness in pre-diabetic patients by this method.

In the present study, fasting stiffness index measured in subjects with pre-diabetic hyperglycemia was significantly higher than that of normoglycemic subjects. However, this trend changed when data were adjusted for other cardiovascular risk factors as potential confounders [Figure 2]. Also, despite significant positive correlations between fasting SI and age, FPG, WC, W/H ratio, SBP and DBP; based on multivariate regression analysis, age and blood pressure (SBP/MAP), but not FPG, or 2-h PG were found as the strong predictors of SI determined in all subjects and in the hyperglycemic group. These findings suggest that the higher DVP-derived SI found in pre-diabetic patients may have resulted from a cluster of frequently accompanied cardiovascular risk factors not merely the glycemic changes. In keeping with this result, Gunarathne et al. reported age and MAP as significant predicators of SI derived from DVP [13]. Also, they found no significant relationship between fasting SI and FPG on multivariate regression analysis. Similarly, a systematic review of 77 published literatures showed that large artery stiffness assessed by PWV has been more significantly associated with age and blood pressure than other risk factors [31]. The results of our study are partially consistent with the findings of Chou et al. [30] who recently reported higher fasting SI derived from DVP in subjects with pre-diabetes mellitus compared to individuals without hyperglycemia. In that study, participants with pre-diabetes mellitus had significantly higher age, WC, SBP and DBP as well as lower HDL-cholesterol compared to participants without hyperglycemia. It is known that the prevalence of hyperglycemia increases with age [32], which is also the main determinant of arterial stiffness [2]. In addition, impaired glucose metabolism has been shown to be strongly associated with other atherosclerotic risk factors including dyslipidemia, hypertension and obesity [33-35]. In the study conducted by Chou and his colleagues no data were provided about the mean SI in the study groups estimated after adjustment for these variables. Thus, the significant difference in DVP-derived SI reported in that study could be also due to other frequently accompanied risk factors rather than just the increased blood glucose at these levels.

According to our results, fasting SI was correlated more strongly with age, while there was a weak relationship between fasting RI and age. In keeping with this, Millasseau et al. [36] suggested SI as a more reliable index for evaluating vascular ageing than RI. Furthermore, Woodman and Watts [11] indicate that the SI derived from DVP analysis provides an estimation of large artery stiffness which is strictly linked to age-related changes.

While differences in fasting and postprandial RI values between the two groups were not statistically significant, they may be important from a clinical view. DVP derived RI is mainly related to the tone of small arteries which can be altered in the presence of hyperglycemia [37]. Moreover, blunted response of RI to endothelium- dependent vasoactive agents has been established as a marker of endothelial dysfunction in individuals with high cardiovascular risk, including those with impaired glucose metabolism [8,15,16,38]. It was shown that endothelium-dependent vasodilation is reduced after an oral glucose loading even in normal individuals [17,18]. To the best of our knowledge, this is the first study investigating the changes in RI in subjects with hyperglycemia following a glucose challenge, using the digital volume pulse analysis technique.

The effect of oral glucose (usually 75 g administrated in a standard oral glucose tolerance test) on endothelial function has been mainly assessed by the change in the flow-mediated vasodilation (FMD) assessment. FMD was shown to decrease within 1 hour after ingestion of the glucose load by healthy subjects as well as patients with IGT and diabetes [19]. However, the change in the post-prandial FMD was more significant in diabetic or impaired glucose tolerant patients, compared to subjects with normal glucose metabolism.

Several mechanisms are thought to account for the temporary impairment of endothelium-dependent vasorelaxation observed following an oral glucose challenge, including generation of oxidative stress and free radicals, a transient loss of NO bioavailability, and a reduction in antioxidant defence systems [17,19,39].

In the present study, a tendency toward a blunted response of DVP-derived parameters (RI) to the oral glucose challenge in subjects with impaired glucose metabolism is consistent with impaired endothelium-dependent vasodilation in these patients, as shown by others in the FMD assessments. In agreement with our results, Gopaul et al. [15] reported a trend toward a reduced change in the RI derived from DVP analysis following inhalation of salbutamol in subjects with impaired glucose tolerance, which became significant in newly diagnosed and established diabetes. Similarly, Chowienczyk et al. [8] showed an attenuated response of RI to inhaled albuterol (salbutamol) in patients with type 2 diabetes mellitus, compared to control subjects. Given the evidence of changes in FMD following an oral glucose challenge in subjects with hyperglycemia as well as the reduction in photoplethysmographic digital volume pulse parameters under salbutamol inhalation, it can be expected that evaluating DVP analysis following an oral glucose challenge could provide additional information in the assessment of vascular function, particularly in newly diagnosed diabetic patients. Further research with a larger sample size is required to extend these encouraging outcomes.

## Conclusion

In the current study, we found age and MAP as the strong predictors of increased arterial stiffness among pre-diabetic subjects. Our findings suggest that the higher DVP-derived SI in patients with pre-diabetic hyperglycemia may result from different frequently accompanied cardiovascular risk factors rather than merely glycemic changes in this range. We also observed a sizeable reduction in postprandial RI that if confirmed in a larger group of individuals it would indicate a derangement of vascular function in hyperglycemia.
